# Correspondence from Paris

**Published:** 1848-07-01

**Authors:** 


### CORRESPONDENCE FROM PARIS.

The state of Paris during the last three months will sufficiently account for the meagre details which are furnished for the investigators into a branch of science demanding quiet research, accurate observation, and the most serious contemplation. Besides this, it has been a matter of considerable difficulty to reach the institutions in which the study of mental disease can only be carried on. The magnificent institutions of the Bicêtre, the Saltpétrière, and of Charenton, are at a considerable distance from the most frequented parts of the town; and there is occasionally some danger in traversing those districts where the discontented populace assemble, not so much from the workmen, as from those whose duty it is to watch them, and who have no scruple in arresting the innocent and curious as well as the turbulent and the mischievous. As the lectures and the visits of the professors take place early in the morning, and as it is necessary to read the newspapers before starting, to ascertain what quarter of the town is most likely to be tranquil, it is almost impossible to reach the lecture-rooms and asylums at a proper period. With regard to medical, or indeed any other but political literature, that is altogether suspended. Nothing is read, and nothing issues from the press, but newspapers, of which one hundred and twenty have started since the 24th of February.

Whilst the revolution has thus affected the promulgation of medical science, it has still more singularly acted upon its followers. The number of physicians who have abandoned their practice to become representatives of the people, has been the subject of much remark. Amongst the ministers now in power are two physicians; one of them, M. Trelat, has held a distinguished position in the lunatic department of the Saltpétrière, and his name is familiar to the student of psychology. There is also a most important fact upon which, hereafter, some curious details may be offered to your readers; the number of persons who, affected by reverse of fortune, by the shocks which they have sustained during the recent events, have become deranged, render the statistics of the public and private asylums of unusual interest, and have unfolded a page in the history of mental disease from which the most important deductions may be drawn. The clinical lectures at the Salpêtrière commenced about a fortnight since; and Dr. Baillarger and M. Lelut opened their respective courses to an unusually large number of pupils, upon whose minds the singular opportunity which the revolution affords of studying insanity seemed to be particularly impressed, for no other branch of medical science has been similarly sought after. The Saltpétrière is an establishment particularly adapted for science. It is, with the exception of the lunatic department, to which females of all ages have access, devoted to females advanced in life; and every woman who has attained the age of seventy-nine has a right to admission. It at present contains five thousand and five persons. It is in itself a small town, beautifully laid out in regular parallel buildings, having shady walks with fine grown avenues interspersed; it is kept in the most perfect order and cleanliness, and is altogether worthy a great nation. Socialism, with all its boasted benevolence, will never raise a more splendid monument than the monarch who founded this institution; and the reveries of Cabet, whose promised colony of Elysium, or "Icaria," for a work of that name has been read with much enthusiasm, will never furnish such a glorious specimen of the power and the will to do good.

From the part of the establishment destined for the reception of lunatics, Dr. Baillarger and M. Lelut select such cases as they think most interesting to the student, analyse the history, dwell upon the symptoms, explain the diagnosis and their prognosis, and then expatiate upon them, so as to enable their auditors to judge of the nature of the treatment, and to watch the progress of the disease. Dr. Baillarger has a European reputation as the editor of the French Journal of Mental Pathology, the *Annales Medico-Psychologique;* he is well known, and his labours highly appreciated; his lectures are clearly and unaffectedly delivered. Having so lately heard him address the Academy of Medicine, I did not go to his introductory lecture; fully persuaded of his powers of teaching, admiring the manners of the physician and the scholar, I did not require to be further convinced that he would do justice to his subject; and remembering the risks I might encounter, I have not attended him this session. But Lelut I had never heard; and therefore determined to make an effort to be present at the first lecture of an individual who has given much interesting information to the medical world; and if he has not gained a leading reputation, has been always regarded as a man of considerable acquirements and deep study. His works best known are *Qu'est ce la Phrenologie?* being an inquiry into the value of psychological systems in general, and that of Gall in particular; *Du Demon de Socrate*—this is a specimen of psychological science applied to history; *L'Amulette de Pascal*—this is a work of singular merit, but little known in England; to those who would study the subject of hallucinations, it affords most valuable materials. Not only does he examine Pascal in all the varied conditions in which he paints himself—his disease, his early genius—but he dives into the recesses of his mind, examines his credulity, details the circumstances at Neuilly with a view to explain them; and then details to us "the tales of truth" that are said to be contained in the hallucinations of Abbé de Brienne and the fiery globe of Benvenuto Cellini.

His work, however, best known in England, at least, oftenest quoted, is, "*Inductions sur la valeur des altérations de l'Encephale dans le delire aiguet dans la folie.*"

M. Lelut delivered his lecture with great ease and fluency; and though his appearance is altogether somewhat too robust for a philosopher, there is quite sufficient of attractive physiognomy to inspire one with confidence in his mental powers. His lecture held forth the promise that he would in the course point out the best steps for the student to follow the science of medical jurisprudence, as far as regarded lunacy; and after having well defined the malady in its different phases, he expatiated upon the view that he entertained, that in most intellectual wanderings there was some leading point which served as a key to explain the disease, and that in many instances, by proper management, you may induce the patient so to dwell upon it, that you may discover, and oftentimes remedy, the cause. Three females were produced to illustrate this position. To one, there were divine revelations made; another had conspiracies formed against her; a third was *enceinte* by the Duke of Orleans; and certainly, by very skilful but apparently trifling questioning, each detailed with great volubility the characteristics by which their malady was marked, and the promise made by the professor was fully borne out.

At the Bicêtre, the great experiment of giving an education to idiots is still going on, and still sanguine hopes are entertained that some of these unfortunate beings may be converted into moral and useful members of society. That the attempt redounds to the honour of those who have undertaken it, all must acknowledge; that some apparent benefit has been the result is an inducement to proceed; and if a few individuals are rescued from the deplorable state to which they were seemingly condemned, humanity will have gained a triumph which ought to be far and widely made known. Even if it lead to the arriving at obtaining absolute distinctions between those whose senses are developed with the utmost difficulty, and after a tedious retardation, and those who are incapable of receiving the rays of intelligence, something will have been gained; but as far as yet I am able to form a judgment, the enthusiastic views of such men as Edouard Seguin will not be confirmed. Delighted with the perusal of his "*Traitement moral, hygiene et education des idiots,*" I had anticipated great results. At present, I must confess myself disappointed, and have laid aside, with something amounting to disgust, the exaggerated works from which I had gleaned my information, and return to such an unpretending work as that of Itard, in which he gives the detail of his watching over the development of the mind of Victor, the wild savage caught in the woods of Aveyron, with increased pleasure, charmed with its unpretending simplicity and truth. What I have seen at the Bicêtre may almost entirely be referred to imitation, and scarcely any cases are much beyond what may be perceived in the monkey genus. Still, however, I would by no means abandon the subject, but would only watch over it with increased vigilance and anxiety, if I did not fear that I might be believed to be a convert to all that is asserted. As soon as my mind arrives at its decision, I shall be too happy to make your journal the medium of the communication of my impressions. The private asylums here are in general under admirable restrictions, and for the most part are humanely conducted. On Sunday last I had occasion to visit the establishment, founded at Ivry by the great and good Esquirol, through the kindness of his nephew, M. Metivier, who, together with Drs. Moreau and Baillarger, carries it upon a scale of great liberality and humanity. I have been allowed to visit occasionally the museum originally founded by Esquirol; it contains a large collection of crania of insane persons, with their history. It forms one of the most interesting chains of evidence I have ever seen. I regret that the museum is unknown to the scientific world, and that such valuable materials are allowed to remain neglected.

I hear of no work, as you may well imagine, either recently published or in the press—indeed, I was grieved to learn from Dr. Baillarger, that he thought that he should for the present suspend the publication of his periodical, which has been of so much use in mental pathology. There has, however, been brought forward a work, not, however, in your immediate department, published at the expense of the Norwegian government, that is exciting much attention. It is entitled, "*Traité de la Spedalskhed, ou Elephantiasis des Grecs, par le Docteur Damelssen et W. Boeck, Professeur de Medécine à Christiana.*" The atlas which accompanies it contains some beautifully executed engravings of the disease, and Doctor Chabert has published a volume entitled "*Des Effets Physiologiques et Therapeutiques des Ethers.*" It is said, that the director of the institution of the deaf and dumb, M. de Lanivoi, is preparing a reply to Seguin's notice of the life of Jacob Rodrigue Pereire, in which, with pertinacity, and apparently on good foundation, he claims all the honours which have been hitherto given to the Abbé de l'Epée, for the education of the deaf and dumb, for the individual whose biography he has so laboriously worked out. Seguin's work is certainly cleverly and elaborately written. That Pereire had a system by which he instructed the deaf and dumb nobody doubts, and that he had a claim prior to the Abbé de l'Epée is equally true; but whilst his invention is admitted, he certainly neither promulgated it nor gave it for the benefit of the world. He alone profited by it, whilst the Abbé de l'Epée, with wonderful industry, laboured for the poor, and received no earthly reward.

*Paris, June* 22, 1848.

